# Diversification of Pakistani Amelogenin-Y-Null Male Haplotypes

**DOI:** 10.1155/2021/5521411

**Published:** 2021-05-04

**Authors:** Nasir Siddique, Ahmad Ali Shahid, Kalsoom Sughra

**Affiliations:** ^1^Department of Biochemistry & Biotechnology, University of Gujrat, Gujrat, Pakistan; ^2^DNA and Serology Section, Punjab Forensic Science Agency, Lahore, Pakistan; ^3^National Centre of Excellence in Molecular Biology, University of the Punjab, Lahore, Pakistan

## Abstract

Amelogenin is a common sex typing marker encountered in forensic case work. Phenotypically normal males have been reported in the literature who exhibit anomalous amelogenin allele. These males express only a single amelogenin peak representing AMEL-X and are called as AMEL-Y-null males. Gender misclassification of such individuals is an obvious consequence of this mutation, as a male sample would falsely appear to be a female sample. This study was aimed to attribute the AMEL-Y-null male DNA profiles encountered in forensic casework in the Pakistani population to appropriate phylogenetic clade based on shared ancestry. A total of 18 null AMEL-Y males were screened out of the sample pool of 5000 male individuals, reflecting mutational frequency of 0.36%. A common phylogenetic ancestor is suggested for 17 individuals, based on computational analysis of the Y-STR haplotypes, shown to be belonging to the J haplogroup while only one sample belonged to the R group. The samples in J groups showed homology with subclades J2b2a M241 and J2b2a PH1648, while R group individual showed 100% homology with R1a. Data are reported after haplotype network development of AMEL-Y-null Pakistani males using Network 10.0 for the study of evolutionary distances and emergence of nodes.

## 1. Introduction

Human gender identification has important applications in forensic casework [1], parentage determination [2], archaeological analysis [3], DNA data basing, and blood sample storage. Sex typing is vital in medical diagnosis of sex-linked diseases and forensic science. Moreover, sex determination is of pronounced significance as additional information in criminal investigations as well as in identification of missing persons, no-suspect cases, and ancient DNA studies. The Y-chromosome sex marker amelogenin is extensively used for determination of chromosomal sex of an unknown individual and differentiating the comparative influences of male and female DNA in the mixed forensic samples [4, 5].

Amelogenin is present on both X and Y chromosomes where it is denoted as AMEL-X and AMEL-Y. AMEL-X is located on the distal short arm of the X chromosome in the p22.1–p22.3 region, while AMEL-Y is present near the centromere of the Y chromosome at p11.2 [6, 7]. There is a small variation between AMEL-X and AMLEY, attributed to a 6 bp deletion in the third intron of the AMEL-X isoform [6, 8, 9]. While this deletion has no phenotypical manifestation, it is, however, conventionally exploited in forensic DNA analysis to differentiate male and female DNA profiles. Many primer sets have been developed for the amplification of the amelogenin gene to use it as a sex typing test [8, 10–12]. The most commonly used primer set executes PCR amplification, resulting in AMEL-X/AMEL-Y amplicons of 106 bp and 112 bp, respectively [1].

The usefulness of the amelogenin marker in forensics has been repeatedly called into question, as many cases of the failure of the amelogenin marker to correctly determine the sex of DNA donors have been reported [13, 14]. Apparently the failure to amplify AMEL-Y denotes the lack of Y-DNA, but, in fact, the Y chromosome is present but the region of the Y chromosome having AMEL-Y is deleted. The reported null AMEL-Y males vary by population, fluctuating from 0.018% to 8%, with the uppermost frequencies of amelogenin failure present in populations of the Indian subcontinent [5]. Mostly, the AMEL-Y-null males reported are due to the fragment deletion in the Y chromosome including AMEL-Y [15, 16].

A Y-chromosomal haplogroup is a cluster or a family of Y chromosomes interrelated by descent or ancestry determined by an explicit set of Y-chromosomal markers. Y haplogroups play a vital role in understanding past migrations and demographic progressions that wrought modern populations [17]. Y-SNP analysis was conventionally conducted for haplogroup designation; however, Y-SNP analyses are time consuming and laborious. New approaches have recently been explored, for example, in silico analysis based on algorithms assignment created with Y-STR profiling data [18–20]. While the accuracy of in silico haplogroup assignment methods was initially under question, a number of recent validation studies including sample groups with both Y-SNP and Y-STR data acclaimed accuracy of more than 95%. The reliability of analysis is further reinforced when using datasets of at least twelve Y-STR loci with more inflexible definite haplogroup assignment thresholds [21–24]. In the forensic perspective, there are adequate Y-STR data accessible that can be explored for population genetics [25–27]. In the current study, we have conducted phylogenetic analysis of the Pakistani AMEL-Y-null males.

## 2. Materials and Methods

### 2.1. Sample Collection

The Punjab Forensic Science Agency (PFSA), Lahore, Pakistan, DNA Database (Bode Match Software) comprises thousands of profiles generated during forensic casework over the years. AMEL-Y-null male individuals search in this database was conducted by filtering out the male individuals with genotype as X, at the amelogenin locus. Eighteen AMEL-Y-null males were identified out of the total male sample pool of 5000. Blood samples of these 18 individuals were aseptically collected with the consent of the donors.

### 2.2. DNA Extraction and Quantification

DNA extraction and purification from the blood samples was carried out by an organic extraction method employing Phenol-Chloroform-Isoamyle alcohol (PCI) extraction protocol followed by nucleic acid purification by ethanol precipitation [28]. Quantification of the DNA extracts was performed on an ABI® Real-Time PCR 7500 system using the Quantifiler® Duo DNA Quantification Kit according to the manufacturer's protocol [29].

### 2.3. STR and Y-Profiling

Y-STR profiling was conducted using an optimal target input DNA (0.5–1.0 ng) using the AmpFlSTR® Y-Filer™ PCR Amplification Kit (Applied Biosystems), at DYS19, DYS385, DYS389I/II, DYS390, DYS391, DYS392, DYS393, DYS437, DYS438, DYS439, DYS448, DYS456, DYS458, DYS635, and Y-GATA-H4 Y-chromosome loci [30]. The manufacturer's recommended protocol was used for the amplification reactions. PCR Amplification of the all samples was carried on the ABI Veriti™ 96 Well Thermal Cycler.

Amplification with the ABI AmpFlSTR® Identifiler® Plus PCR Amplification Kit and ABI Globalfiler™ was also carried out for the null samples following recommendations by the manufacturer [31, 32]. Positive and negative amplification controls were used for evaluating the efficiency of the amplification.

The amplified products were electrophoretically separated on the ABI 3500 series Genetic Analyzer (Applied Biosystems) using an injection time of 5 seconds at 3 kV, whilst POP-4 TM, GeneScan 600 LIZ® Size Standard, loading mix, and other reagents were used according to the manufacturer's instructions. Genotyping results were analyzed using GeneMapper® ID-X software version 1.4 [33].

### 2.4. Haplogroup Prediction

The haplotypes of the AMEL-Y-null Pakistani males sample obtained through Y-STR profiling were submitted to the web-based software Nevgen Y-DNA haplogroup predictor (http://www.nevgen.org). Nevgen is a typical interpreter which predicts haplogroup based on STR profiles. Predicted haplogroup, fitness score, and probability were calculated for all the samples under study. Haplotypes comprising 17 Y-STR loci of all the samples were uploaded to Nevgen. Haplogroup was assigned to the samples on the basis of highest probability scores [20].

### 2.5. Statistical Analysis

The genetic relationship between haplotypes of AMEL-Y-null Pakistani males was analyzed using NETWORK 10.0 software (http://www.fluxusengineering.com). Median joining methods were employed using the frequency >1 criterion with active external rooting and MJ square [34–36]. For network construction, the duplicated locus DYS385 was not used since the constituent loci are not distinguished in this assay.

Time estimation was performed using a default mutation rate of 01 mutation per 20180 years in NETWORK 10.0 software. Using SEQ-1 arbitrarily as an ancestral node, time was estimated for each AMEL-Y-null male SEQ group as descendent node separately as well as with all sequences.

Forward simulation was carried out using the default setting of a NETWORK 10.0 mutation rate of 01 mutation per 20180 years, maximum population decrease/increase per generation at 50 and 1000 number of population equally distributed. Mismatch mutation rate was also determined using the same software.

## 3. Results and Discussion

### 3.1. AMEL-Y-Null Males in the Pakistani Population

Even though amelogenin is the workhorse of DNA sex determination for decades, the failures of this marker have been reported continuously from different populations. Expansion in CODIS core loci in the USA was proposed [37], and 20 loci are now categorized as core CODIS loci since 2017. The new commercial kits include additional autosomal STR loci and a Y-STR locus to help in sex determination in case amelogenin fails to amplify.

**Figure 1:**
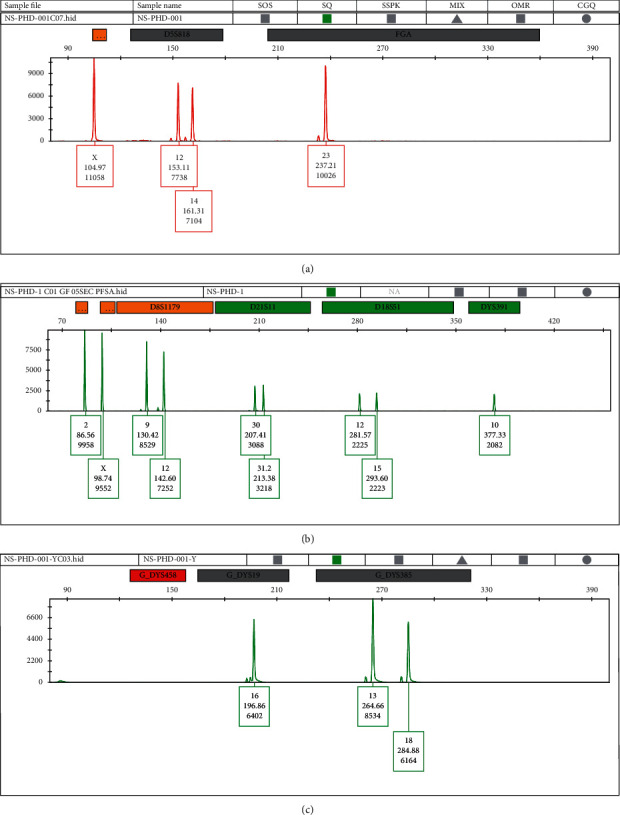
A representative STR electropherogram of AMEL-Y-null Pakistani males. (a) Panel of STR profile obtained using the Identifiler Plus STR system showing the absence of Amel-Y. (b) Panel of STR profile obtained using the Global Filer STR system showing the absence of Amel-Y. (c) Panel of haplotype profile obtained using the Y-filer STR system showing the absence of DYS458.

The local database of PFSA, Lahore, Pakistan, was explored for the DNA profiles of male reference samples incorrectly genotyped as females. Eighteen male individuals showed a female genotype, depicted by the nonappearance of the 112 bp AMEL-Y peak (Figure 1(a)). The frequency of the AMEL-Y-null allele was calculated as 0.36% (18/5000) in the Pakistani population. These samples were labeled as SQ-1–SQ-18. The frequency of AMEL-Y-null males in various populations has been studied and has been shown to vary in different populations ranging from 0.037 to 10% [38–42].

The AMEL-Y-null males who showed amelogenin-Y amplification failure with the Identifiler plus kit also showed the AMEL-Y negative results with Globalfiler™; however, all eighteen samples were typed as male correctly due to the presence of DYS391 and Y-InDel loci in the Globalfiler kit (Figure 1(b)). This concordance of AMEL-Y null in both PCR systems ruled out the possibility of point mutation in the primer binding site as both use different sets of primers for AMEL amplification. These results, coupled with the finding that DYS458 did not amplify with the Y-filer kit, suggested that there is a fragment deletion in the p arm of the Y chromosome in these eighteen individuals (Figure 1(c)).

Subsequent amplification of the 16 Y-filer™ Y-STR loci gave additional conclusive results that these 18 samples are from true male individuals.

### 3.2. Haplotypes of AMEL-Y-Null Males

The Y-STR profiles of null AMEL-Y males share a maximum of fifteen alleles and a minimum of only one allele, i.e., SQ-8. The incredibly analogous haplotypes of 5 samples (sharing of maximum 15 alleles) indicated the possibility of similar phylogenetic origin. The amelogenin-Y dropout in all eighteen Pakistani male samples was accompanied by the same pattern of insertion at the Y-InDel locus showing genotype 2 at this locus, and a genotype of DYS391 was observed as 10 in all samples except for sample SQ-8 and SQ-17 that showed genotype 11 at this locus.

The haplotypes of all the Pakistani AMEL-Y negative males showed the deletion at DYS458 while all other alleles at different loci were correctly genotyped. These findings further strengthened the concept of having fragment deletion in the Y chromosome of these individuals. Three different deletion patterns of AMEL-Y-null cases have been reported in literature: AMEL-Y-DYS456, AMEL-Y-DYS458, and AMEL-Y-DYS458-DYS522 [42–44]. The deletion observed in all eighteen AMEL-Y-null male samples of the Pakistani population was found to be AMEL-Y and DYS458. The haplotypes of AMEL-Y-null Pakistani males are enlisted in Table 1.

**Figure 2:**
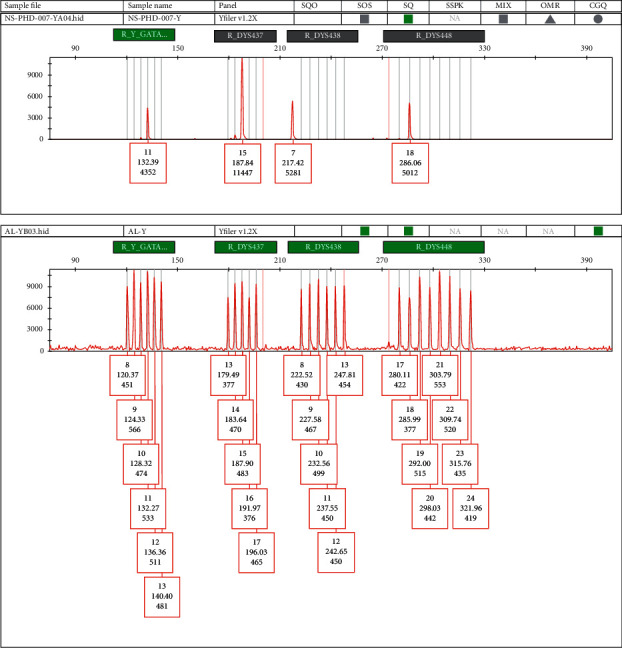
A profile of AMEL-Y-null male with off-ladder allele variant 7 at DYD438.

Despite few dissimilarities, most of the Y STR loci in AMEL-Y-null males have the similar haplotypes suggesting that these deletions may possibly have occurred in the same paternal lineage [45]. Five of the samples (SQ 3, 4, 5, 9, and 11) show identical haplotypes. Another group (SQ 7, 14, 16, and 17) shares the same Y haplotype. An off-ladder allele variant was observed at DYS438 in the haplotypes of SQ 7, 14, 16, and 17 which was calculated as 7 (Figure 2). The electropherograms of these samples are illustrated in Figure S1. SQ-8 has a unique versatile haplotype among the eighteen AMEL-Y-null Pakistani males.

### 3.3. Haplogroups of AMEL-Y-Null Males

**Figure 3:**
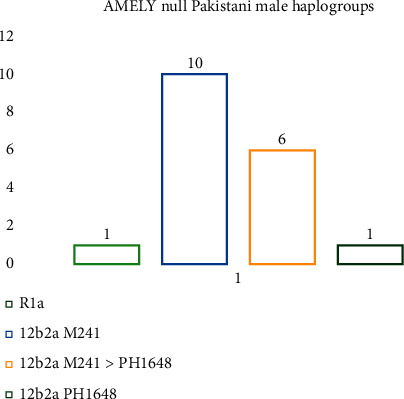
Graphical representation of relative abundance of different haplogroups observed in AMEL-Y-null Pakistani males.

Haplogroup and subclades were predicted using the Nevgen Y-DNA haplogroup predictor for all eighteen AMEL-Y-null males using their Y STR haplotypes. SQ-1 showed the highest probability value of 99.7% with J group and subclade J2b2a M241. Seventeen out of eighteen AMEL-Y-null males have the Y-STR profiles which showed the maximum probability with haplogroup J while only one (SQ-8) showed 100% homology with the R group (Figure 3).

Y-DNA haplogroup J evolved in the ancient Near East and was carried into North Africa, Europe, Central Asia, Pakistan, and India. J haplogroup ancestors spread in the Fertile Crescent (Middle East area). Middle Eastern traders brought this genetic marker to the Indian subcontinent [46, 47]. Y-DNA haplogroup R-M207 is ascended approximately 27,000 years ago in Asia, having two subclades R1 and R2. In India and Pakistan, this is one of the prevalent haplogroups [48]. Detailed probability values of AMEL-Y-null Pakistani males with different haplogroups and subclades as determined through Nevgen Y-DNA haplogroup predictor are given in Table S1.

### 3.4. Statistical Analysis

**Figure 4:**
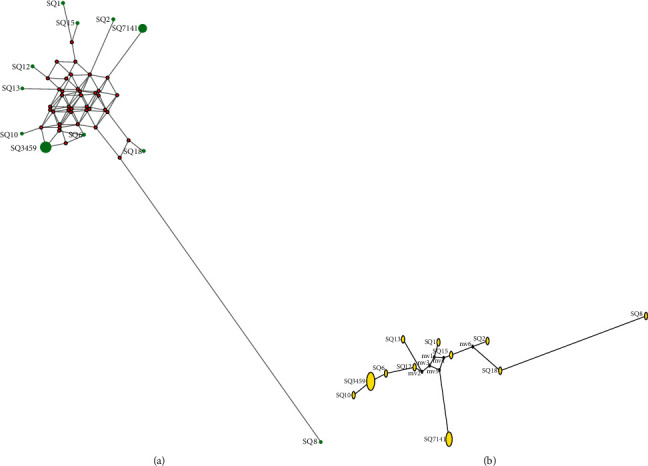
YSTR haplotype network (a) and phylogenetic tree (b) of AME-Y-null Pakistani males drawn using the median joining method.

The median joining method was used for the network preparation using NEWTWORK 10.0 software to study the genetic relationships between haplotypes of AMEL-Y-null Pakistani males (Figure 4(a)). The phylogenetic tree showed that SQ-8 is genetically distant from other AMEL-Y-null Pakistani males (Figure 4(b)).

The total number of mutations disregarding the torso is 49, estimated number of mutations of the shortest tree within the torso is 3, and estimated number of mutations of the shortest tree is 52.

The statistical data for this phylogenetic analysis showed that the total number of mutations disregarding the torso is 49, estimated number of mutations of the shortest tree within the torso is 3, and estimated number of mutations of the shortest tree is 52. The number of mutations at each genetic locus was observed except DYS392. Maximum five mutations were present at DYS389IIab (Table S2).

**Figure 5:**
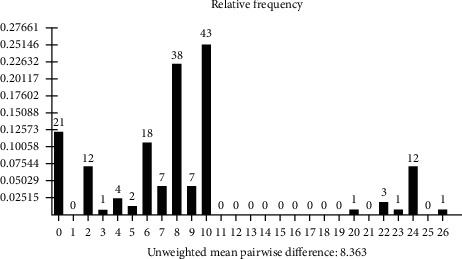
Mismatch frequency of AMEL-Y-null Pakistani male haplotypes.

Forward simulation was performed using a sequence length of 369 with the number of generations 1000 and mutation rate of one mutation every 20180 years. Pairwise mismatch frequency was calculated using NETWORK 10.0. The unweighted mean pairwise difference was observed to be 8.363.  Figure 5 demonstrates the relationship between pairwise differences and their relative frequencies among AMEL-Y-null males in the Pakistani population.

Default mutation rate of one mutation per 20180 was used for time estimation in years and Rho.

Time estimation was carried out using the default mutation rate of 01 mutation per 20180 years [49] in NETWORK 10.0 software. The maximum time difference was observed in the SQ-1–SQ-8 302,700 years and 15 mutations (Rho) (Table 2).

## 4. Conclusions

The current study of 18 AMEL-Y-null males out of a total of 5000 male reference samples with the frequency of 0.36% in Pakistani populations emphasizes the requirement for the employment Y-STR markers for sex typing to acquire accurate results. Dropout of DYS458 in all samples and high frequency of Y-STR allele sharing (up to 15 alleles in 5 cases) reflect an overall phylogenetic ambiance amongst them. Y-chromosome fragment deletion was implied on the basis of simultaneous null allele finding at AMEL-Y and DYS458. A majority of the AMEL-Y-null males in the Pakistani population showed the maximum homology with haplogroup J2b2a. Phylogenetic analysis demonstrated that one individual SQ-8 is far different from other seventeen AMEL-Y-null males involved in the study.

## Figures and Tables

**Table 1:** Y-STR haplotypes of AMEL-Y-null males in the Pakistani Population. The off-ladder allele variant 7 observed is highlighted by the shaded cell of the table.

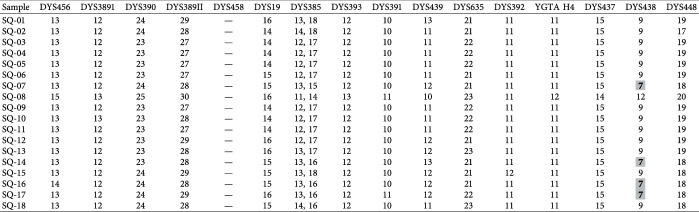

**Table 2:** Time estimation of mutation from SQ-1 to different variants (SQ-2 to SQ-18) of AMEL-Y-null male Pakistani individuals in Rho and years.

Sr. No.	Sample	Age in mutation (Rho)	Standard deviation (sigma)	Age in years	Standard deviation in years
1	2	4	0.70711	80,720	14269.415
2	3, 4, 5, 9	8.57	1.2122	172,971	24467.854
3	6	4	0.86603	80,720	17476.393
4	7, 14, 16, 17	8	1.9596	161,440	39544.562
**5**	**8**	**15**	**2.2361**	**302,700**	**45123.851**
6	10	6	0.70711	121,080	14269.415
7	12	2	0.5	40,360	10090
8	13	3	0.70711	60,540	14269.415
9	15	1.5	0.70711	30,270	14269.415
10	18	5	1	100,900	20180
11	Overall	9.5263	1.9502	192,241	39355.264

## Data Availability

Data are available on reasonable request.

## Supplementary Materials

Supplementary Materials Figure S1: electropherograms of AMEL-Y-null male with off-ladder allele variant 7 at DYD438 (SQ 7, 14, 16, and 17). Table S1: haplogroups of AMEL-Y-null Pakistani males determined through web-based software Nevgen Y-DNA haplogroup predictor. Table S2: maximum number of mutations at each character studies in haplotypes of AMEL-Y-null Pakistani males. Two characters are appended to the locus name (e.g., “DYS389II”) to distinguish between repeat numbers (e.g., “aa” = 27 repeats, “ab” = 28 repeats).
