# Supplementary material for: Diversification of Pakistani Amelogenin-Y-Null Male Haplotypes
Source: Scientifica (Cairo). 2021 May 4;2021:5521411. doi: 10.1155/2021/5521411 (PMC8116151; doi:10.1155/2021/5521411)
Supplement: Supplementary Materials — Figure S1: electropherograms of AMEL-Y-null male with off-ladder allele variant 7 at DYD438 (SQ 7, 14, 16, and 17). Table S1: haplogroups of AMEL-Y-null Pakistani males determined through web-based software Nevgen Y-DNA haplogroup predictor. Table S2: maximum number of mutations at each character studies in haplotypes of AMEL-Y-null Pakistani males. Two characters are appended to the locus name (e.g., “DYS389II”) to distinguish between repeat numbers (e.g., “aa” = 27 repeats, “ab” = 28 repeats). [file 5521411.f1.docx]

**Supplementary Material**

Figure S1: Electropherograms of AMEL-Y null male with off ladder allele variant 7 at DYD438 (SQ 7, 14, 16 and 17).

**
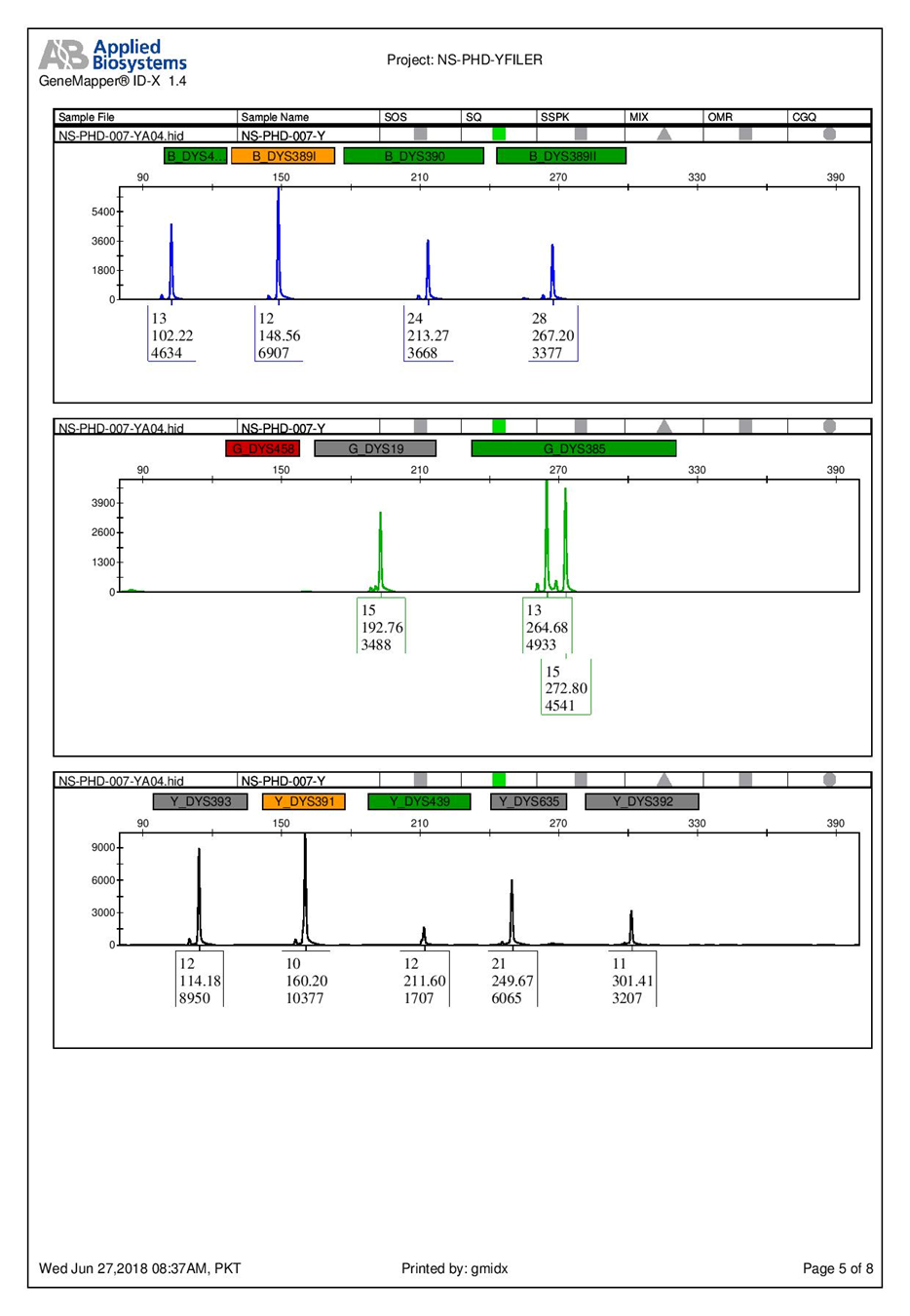

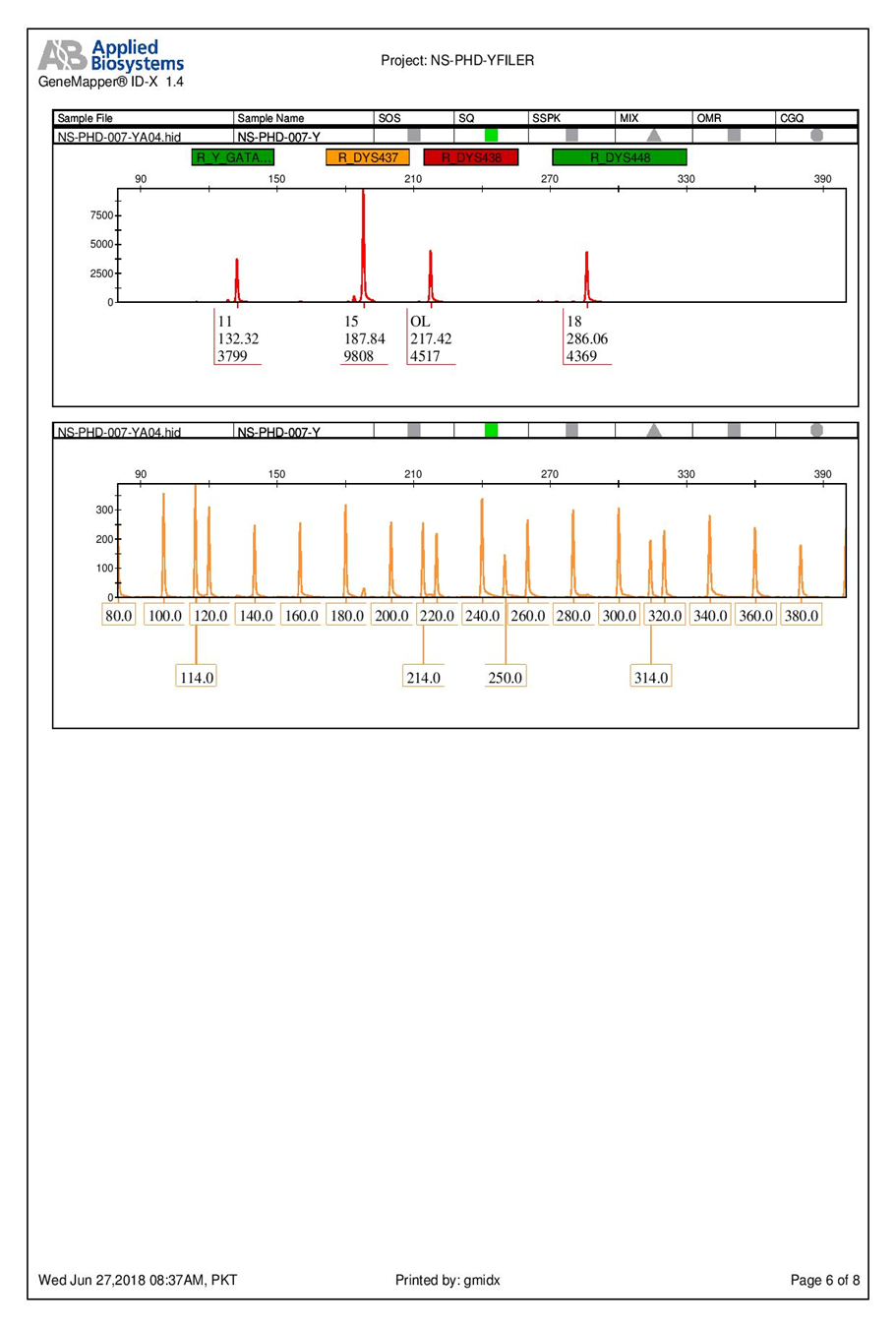
**

**
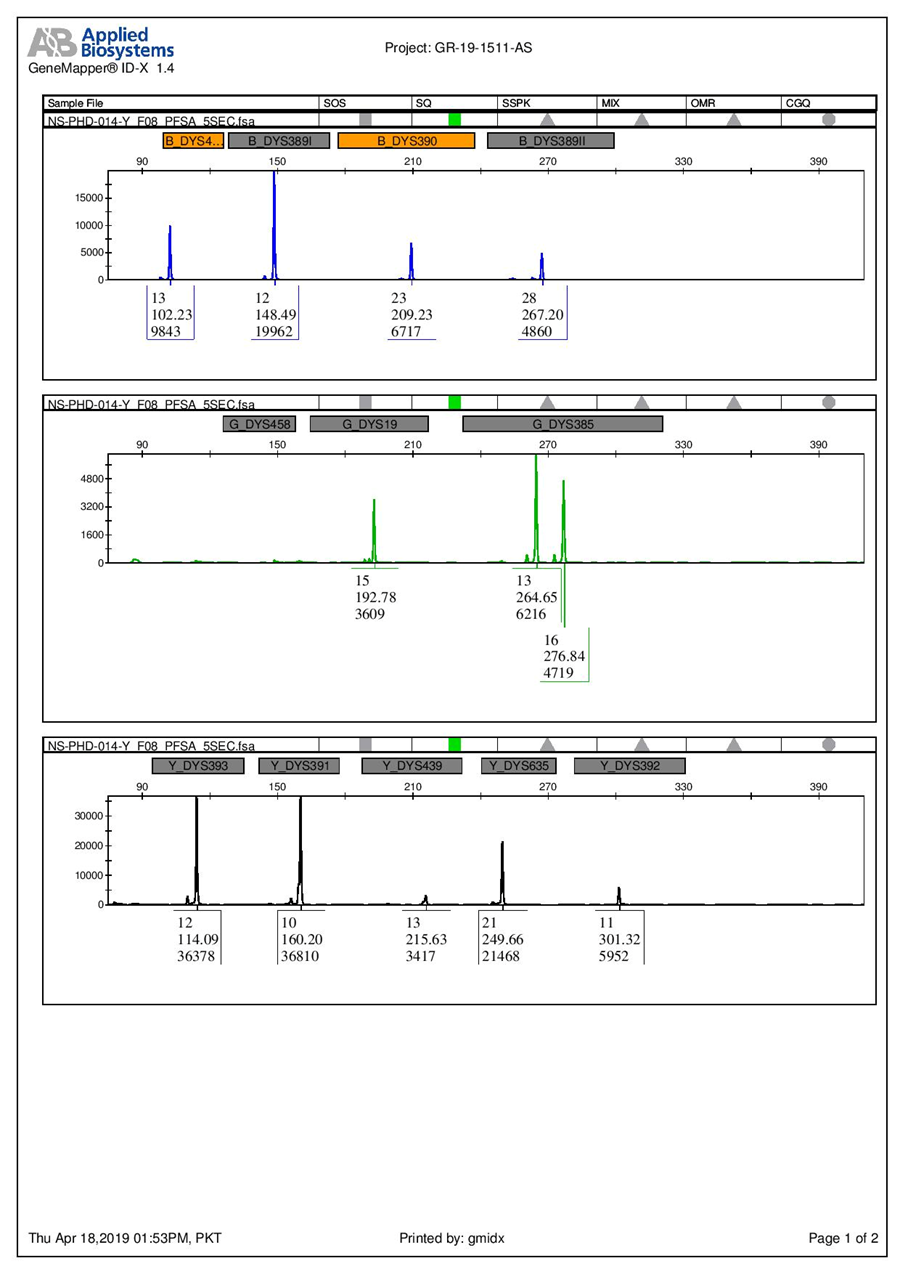

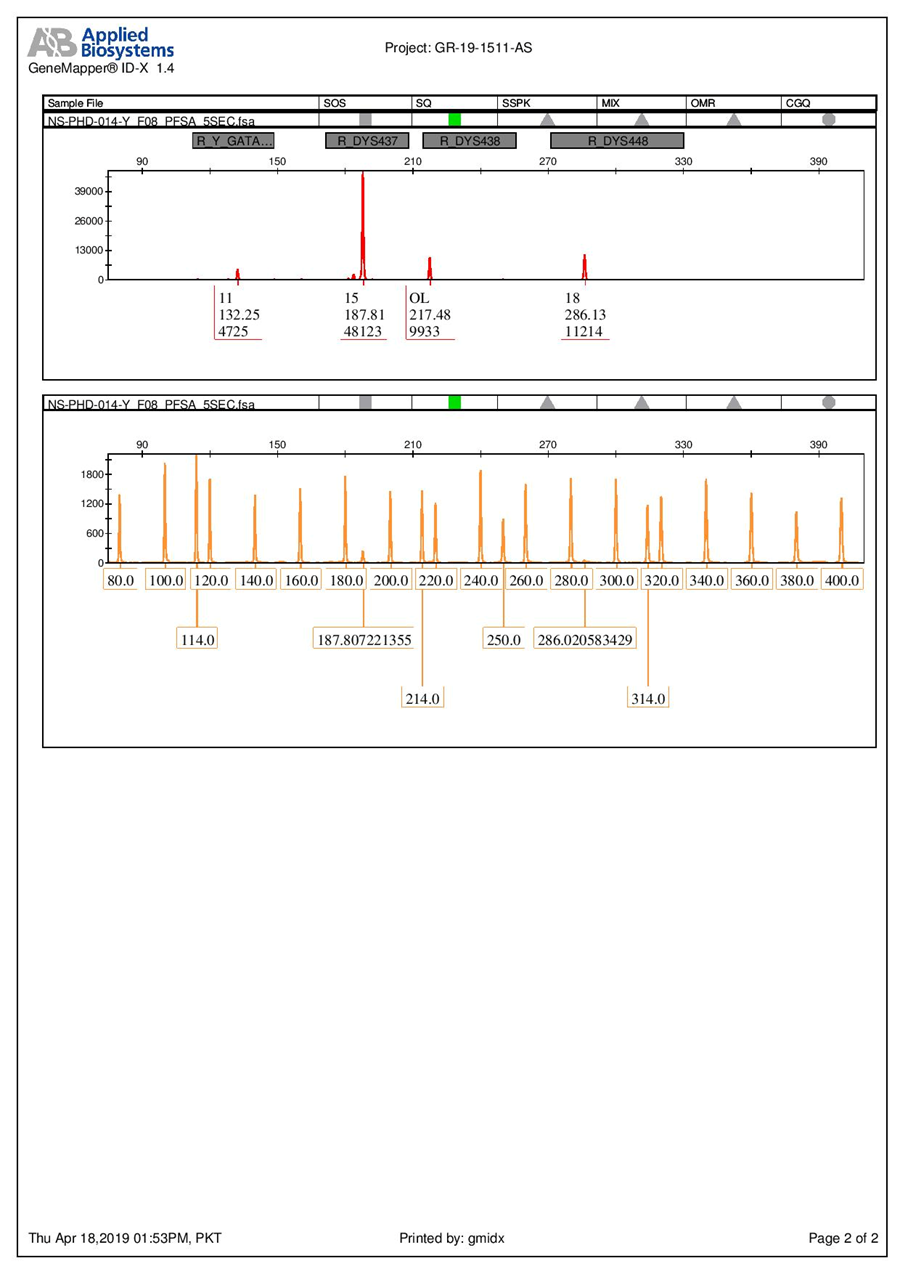

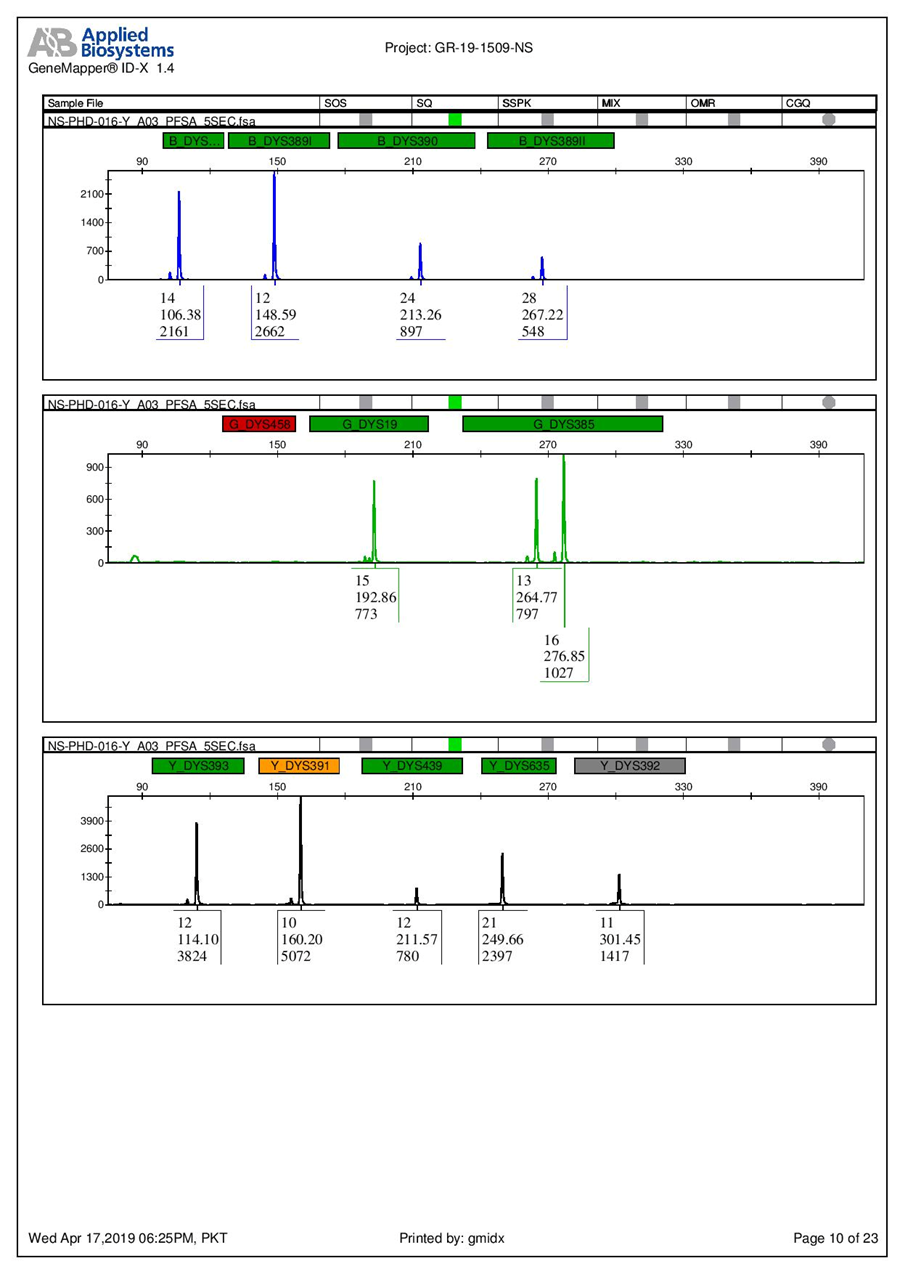

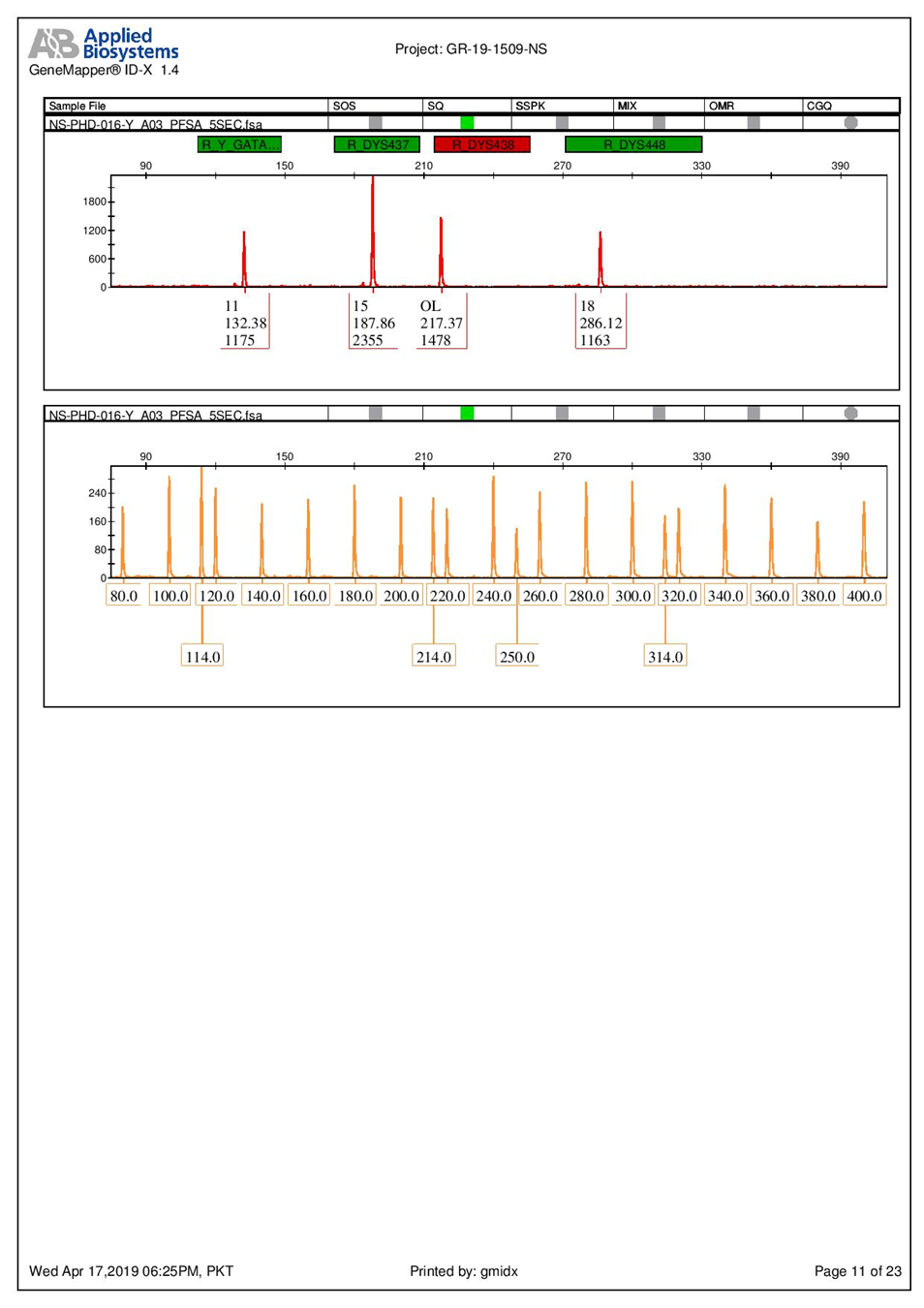

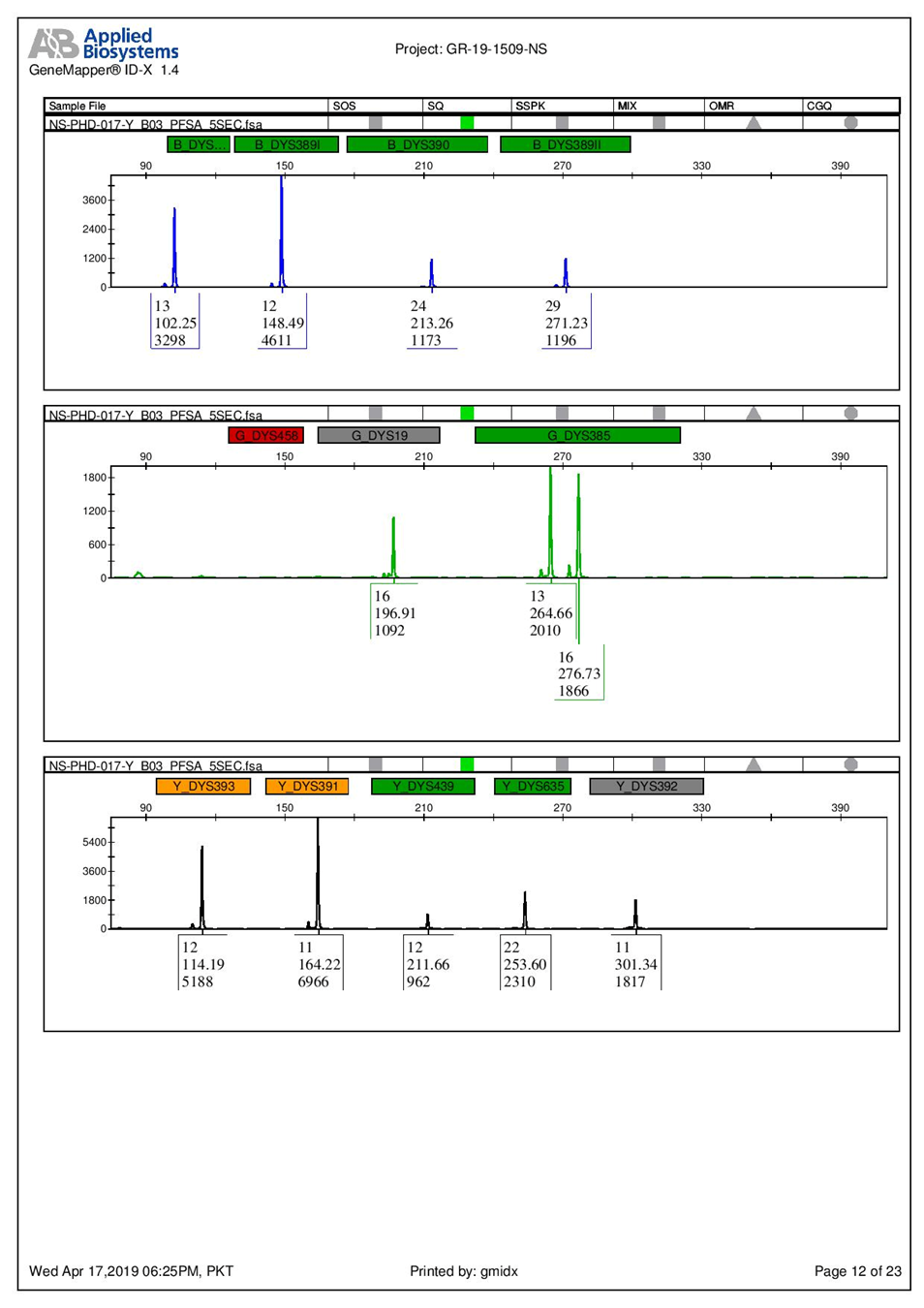

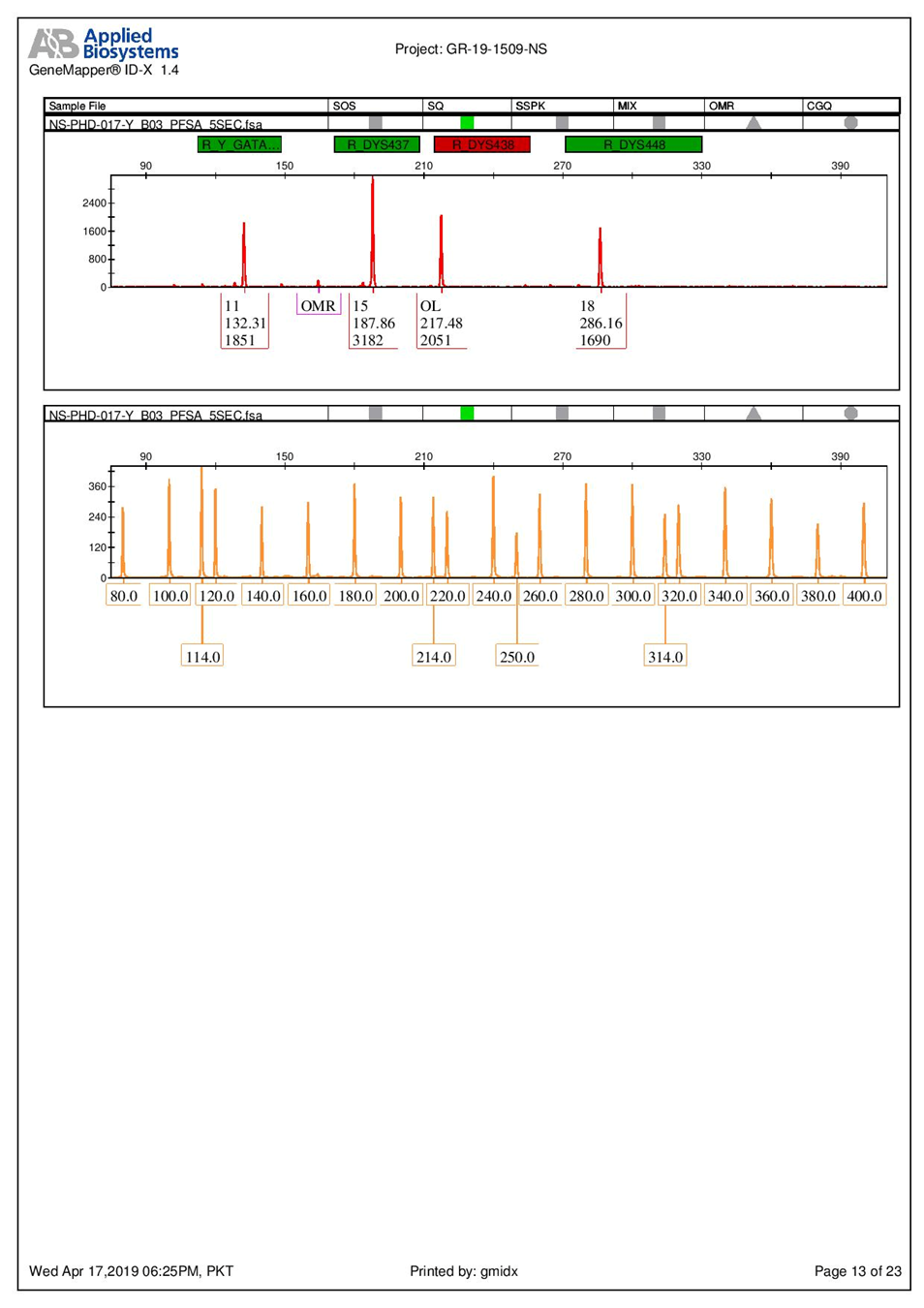
**

Table S1: Haplo groups of AMELY null Pakistani Males determined through web based software Nevgen Y-DNA haplogroup predictor

| Sample No. | Halpo Group | Clade | Subclade(S) | Probability %age | Unsupported subclade |
| --- | --- | --- | --- | --- | --- |
| 8 | **R** | R1a | - | 100 | - |
| 1 | **J** | J2b2a | M241 | 99.7 | - |
| 13 |  | J2b2a | M241 | 99.2 | - |
| 15 |  | J2b2a | M241 | 99.2 | - |
| 2 |  | J2b2a | M241 | 98.6 | - |
| 18 |  | J2b2a | M241 | 98.2 | - |
| 6 |  | J2b2a | PH1648 M241 | 96.2  3.8 | - |
| 12 |  | J2b2a | M241 | 87.14 | 9% |
| 7 |  | J2b2a | M241 | 65.3 | 34.6 |
| 14 |  | J2b2a | M241 | 65.3 | 34.6 |
| 16 |  | J2b2a | M241 | 65.3 | 34.6 |
| 17 |  | J2b2a | M241 | 65.3 | 34.6 |
| 3 |  | J2b2a | M241 PH1648 | 53.2 43.8 | - |
| 4 |  | J2b2a | M241 PH1648 | 53.2 43.8 | - |
| 5 |  | J2b2a | M241 PH1648 | 53.2 43.8 | - |
| 9 |  | J2b2a | M241 PH1648 | 53.2 43.8 | - |
| 10 |  | J2b2a | M241 PH1648 | 53.2 43.8 | - |
| 11 |  | J2b2a | M241 PH1648 | 53.2 43.8 | - |

Table S2: Maximum number of mutations at each character studies in haplotypes of AMEL-Y null Pakistani Males. Two characters are appended to the locus name (e.g. " DYS389II ") to distinguish between repeat-numbers (e.g. "aa"=27 repeats, "ab"=28 repeats)

| No. of Mutations | Y STR Locus |
| --- | --- |
| 5 | DYS389IIab |
| 4 | DYS19ab |
| 3 | DYS635aa , DYS385Bad |
| 2 | DYS19aa , DYS389Iaa , DYS389IIaa , DYS439ab ,  DYS385Bab , DYS385Bac , DYS385Aab , DYS385Aac , DYS635ab |
| 1 | DYS439ac , DYS448aa , DYS438aa , DYS438ab ,  DYS456aa , DYS456ab , DYS390ab , DYS389IIac ,  DYS385Aaa , DYS385Baa , DYS393aa , DYS391aa,  DYS439aa , DYSH4aa , DYS437aa , DYS438ac ,  DYS438ad , DYS438ae , DYS448ab , DYS390aa |
